# Supplementary material for: Biopsy-Controlled Non-Invasive Quantification of Collagen Type VI in Kidney Transplant Recipients: A Post-Hoc Analysis of the MECANO Trial
Source: J Clin Med. 2020 Oct 7;9(10):3216. doi: 10.3390/jcm9103216 (PMC7600059; doi:10.3390/jcm9103216)
Supplement: Supplementary file 1 [file jcm-09-03216-s001.pdf]

Journal of
Clinical Medicine

MDPI

Supplemental material

Biopsy-Controlled Non-Invasive Quantification of Collagen Type VI in Kidney Transplant Recipients: A Post-hoc Analysis of the MECANO Trial

Manuela Yepes-Calderón[1,§], Camilo G. Sotomayor[1,§,*], Daniel Guldager Kring Rasmussen[2], Ryanne S. Hijmans[1], Charlotte A. te Velde-Keyzer[1], Marco van Londen[1], Marja van Dijk[1], Arjan Diepstra[3], Stefan P. Berger[1], Morten Asser Karsdal[2], Frederike J. Bemelman[4], Johan W. de Fijter[5], Jesper Kers[6,7,8], Sandrine Florquin[6,7], Federica Genovese[2], Stephan J.L. Bakker[1], Jan-Stephan Sanders[1], Jacob van den Born[1]

[1]Division of Nephrology, Department of Internal Medicine, University Medical Center Groningen, University of Groningen, Groningen, The Netherlands
[2]Nordic Bioscience A/S, Herlev, Denmark
[3]Department of Pathology and Medical Biology, University Medical Center Groningen, University of Groningen, Groningen, The Netherlands
[4]Department of Nephrology, Amsterdam University Medical Center, University of Amsterdam, Amsterdam, The Netherlands
[5]Department of Nephrology, Leiden University Medical Center, University of Leiden, Leiden, The Nether lands
[6]Amsterdam UMC, University of Amsterdam, Department of Pathology, Amsterdam Institute for Infection and Immunity (AII), Amsterdam Cardiovascular Sciences (ACS), Amsterdam, The Netherlands
[7]Leiden University Medical Center, Department of Pathology, Leiden Transplant Center, Leiden, The Netherlands
[8]Van 't Hoff Institute for Molecular Sciences (HIMS), University of Amsterdam, Amsterdam, The Netherlands

§ These authors contributed equally to this work
* Correspondence: c.g.sotomayor.campos@umcg.nl; Tel.: 0031 061 921 08 81

Received: date; Accepted: date; Published: date

1. **Supplemental table of content**

Table S1. Biomarkers and histological characteristics during follow-up on KTRs by primary kidney disease.…………………………………..………………………….. Page 3

Table S2. Biomarkers and histological characteristics during follow-up on KTRs by primary kidney disease assuming all patients with unknown cause would have had glomerulonephritis as primary kidney disease.…………………………………………………………………………..Page 4

Table S1. Biomarkers and histological characteristics during follow-up on KTR by primary kidney disease

Biomarkers and histological characteristics	Primary kidney disease			*P*
	Polycystic kidney disease	Glomerulonephritis	Hypertension	
n	**24**	**16**	**15**	
**Biomarkers**				
**6-months**				
Plasma				
PRO-C6 (ng/mL)[a]	10 (4.7)	8.7 (2.3)	9.7 (2.5)	0.85
Urine				
PRO-C6 (ng/mg creat)[b]	6.8 (5.0-24.1)	7.2 (3.5-16.3)	3.9 (2.9-7.5)	0.99
**24-months**				
Plasma				
PRO-C6 (ng/mL)[c]	10.3 (5.1)	9.3 (4.9)	9.3 (2.0)	0.99
Urine				
PRO-C6 (ng/mg creat)[b]	5.9 (4.3-29.2)	7.4 (4.5-14.9)	6.0 (3.3-21.0)	0.58
**Delta$_{24-6}$**				
Plasma				
PRO-C6 (ng/mL)[c]	0.2 (5.9)	-0.77 (2.7)	-0.29 (2.1)	0.83
Urine				
PRO-C6 (ng/mg creat)[b]	-0.6 (-2.1-4.8)	1.1 (-2.5 5.5)	0.2 (-1.0-3.1)	0.31
**Histological analyses**				
**6-months**				
IF/TA-score	1.0 (0.0-1.0)	1.0 (0.0-1.0)	1.0 (1.0-1.0)	0.51
PSR, %	13.0 (6.7)	13.9 (5.7)	11.9 (6.6)	0.92
ti-score, %	10.0 (5.0-16.0)	9.0 (3.0-18.8)	10.0 (5.0-20.0)	0.55
**24-months**				
IF/TA-score	1.0 (1.0-1.8)	1.0 (1.0-1.8)	1.0 (1.0-2.0)	0.83
PSR, %	16.2 (7.5)	16.5 (11.6)	17.3 (13.2)	0.27
ti-score, %	20.0 (10.0-30.0)	10.0 (8.5-43.8)	15.0 (8.0-60.0)	0.17
**Delta$_{24-6}$**				
IF/TA-score	0.5 (0.0-1.0)	0.5 (0.0-1.0)	0.0 (0.0-1.0)	0.92
PSR, %	3.17 (7.5)	2.6 (12.1)	5.5 (13.7)	0.25
ti-score, %	10 (0-20)	2.5 (-5.75-16.8)	5.0 (-2.0-30.0)	0.20

Data available in [a]73, [b]62, [c]36 patients. CsA, cyclosporine group; EVL, everolimus group; PRO-C6, released pro-peptide of collagen type VI (endotrophin); IF/TA, interstitial fibrosis/tubular atrophy; PSR, Picro Sirius Red; ti-score, total inflammation score.

Table S2. Biomarkers and histological characteristics during follow-up on KTR by primary kidney disease assuming all patients with unknown cause would have had glomerulonephritis as primary kidney disease.

Biomarkers and histological characteristics	Primary kidney disease			*P*
	Polycystic kidney disease	Glomerulonephritis	Hypertension	
n	**24**	**32**	**15**	
**Biomarkers**				
**6-months**				
Plasma				
PRO-C6 (ng/mL)[a]	10 (4.7)	8.6 (3.4)	9.7 (2.5)	0.77
Urine				
PRO-C6 (ng/mg creat)[b]	6.8 (5.0-24.1)	7.95 (4.86-19.07)	3.9 (2.9-7.5)	0.99
**24-months**				
Plasma				
PRO-C6 (ng/mL)[c]	10.3 (5.1)	9.2 (5.1)	9.3 (2.0)	0.98
Urine				
PRO-C6 (ng/mg creat)[b]	5.9 (4.3-29.2)	7.4 (4.0-17.5)	6.0 (3.3-21.0)	0.58
**Delta$_{24-6}$**				
Plasma				
PRO-C6 (ng/mL)[c]	0.2 (5.9)	1.07 (3.4)	-0.29 (2.1)	0.80
Urine				
PRO-C6 (ng/mg creat)[b]	-0.6 (-2.1-4.8)	1.2 (-2.7-14.9)	0.2 (-1.0-3.1)	0.31
**Histological analyses**				
**6-months**				
IF/TA-score	1.0 (0.0-1.0)	1.0 (0.0-1.0)	1.0 (1.0-1.0)	0.41
PSR, %	13.0 (6.7)	13.4 (6.0)	11.9 (6.6)	0.87
ti-score, %	10.0 (5.0-16.0)	10.0 (5.0-15.0)	10.0 (5.0-20.0)	0.55
**24-months**				
IF/TA-score	1.0 (1.0-1.8)	1.0 (1.0-2.0)	1.0 (1.0-2.0)	0.79
PSR, %	16.2 (7.5)	17.3 (9.8)	17.3 (13.2)	0.20
ti-score, %	20.0 (10.0-30.0)	11.5 (8.5-38.3)	15.0 (8.0-60.0)	0.19
**Delta$_{24-6}$**				
IF/TA-score	0.5 (0.0-1.0)	1.0 (0.0-1.0)	0.0 (0.0-1.0)	0.88
PSR, %	3.17 (7.5)	3.8 (11.5)	5.5 (13.7)	0.19
ti-score, %	10 (0-20)	5.0 (-1.5-21.5)	5.0 (-2.0-30.0)	0.20

Data available in [a]73, [b]62, [c]36 patients. CsA, cyclosporine group; EVL, everolimus group; PRO-C6, released pro-peptide of collagen type VI (endotrophin); IF/TA, interstitial fibrosis/tubular atrophy; PSR, Picro Sirius Red; ti-score, total inflammation score.
